# Physiotherapy with integrated virtual reality for patients with complex chronic low back pain: protocol for a pragmatic cluster randomized controlled trial (VARIETY study)

**DOI:** 10.1186/s12891-023-06232-0

**Published:** 2023-02-20

**Authors:** Syl Slatman, Raymond Ostelo, Harry van Goor, J. Bart Staal, Jesper Knoop

**Affiliations:** 1 Research Group for Musculoskeletal Rehabilitation, HAN University of Applied Sciences, Nijmegen, the Netherlands; 2 Biomedical Signals and Systems Group, Faculty of Electrical Engineering, Mathematics and Computer Science, University of Twente, Enschede, the Netherlands; 3 Department of Health Sciences, Faculty of Science and Amsterdam Movement Science Research Institute, Vrije Universiteit, Amsterdam, the Netherlands; 4 Department of Epidemiology and Data Science, Amsterdam UMC location Vrije Universiteit & Amsterdam Movement Sciences, Musculoskeletal Health, Amsterdam, the Netherlands; 5 Department of Surgery, Radboud University Medical Center, Nijmegen, the Netherlands; 6 Radboud Institute for Health Sciences, IQ healthcare, Radboud University Medical Center, Nijmegen, the Netherlands

**Keywords:** Virtual reality (VR), Physiotherapy, Chronic low back pain, Cluster randomized controlled trial (RCT), Study protocol

## Abstract

**Background:**

Chronic low back pain (CLBP) is the most common chronic pain condition worldwide. Currently, primary care physiotherapy is one of the main treatment options, but effects of this treatment are small. Virtual Reality (VR) could be an adjunct to physiotherapy care, due to its multimodal features. The primary aim of this study is to assess the (cost-)effectiveness of physiotherapy with integrated multimodal VR for patients with complex CLBP, compared to usual primary physiotherapy care.

**Methods:**

A multicenter, two-arm, cluster randomized controlled trial (RCT) including 120 patients with CLBP from 20 physiotherapists will be conducted. Patients in the control group will receive 12 weeks of usual primary physiotherapy care for CLBP. Patients in the experimental group will receive treatment consisting of 12 weeks of physiotherapy with integrated, immersive, multimodal, therapeutic VR. The therapeutic VR consists of the following modules: pain education, activation, relaxation and distraction. The primary outcome measure is physical functioning. Secondary outcome measures include pain intensity, pain-related fears, pain self-efficacy and economic measures. Effectiveness of the experimental intervention compared to the control intervention on primary and secondary outcome measures will be analyzed on an intention-to-treat principle, using linear mixed-model analyses.

**Discussion:**

This pragmatic, multicenter cluster randomized controlled trial, will determine the clinical and cost-effectiveness of physiotherapy with integrated, personalized, multimodal, immersive VR in favor of usual physiotherapy care for patients with CLBP.

**Trial registration:**

This study is prospectively registered at ClinicalTrials.gov (identifier: NCT05701891).

## Background

Low back pain (LBP) is the principal cause of global disability and accompanied by high health care utilization and societal costs [1, 2]. Chronic low back pain, defined as back pain that persists for 3 months or longer [3], is the most common chronic pain condition worldwide [4].

Primary care physiotherapy is currently one of the main treatment options for CLBP, mostly consisting of pain education and exercise therapy [5]. Unfortunately, the effectiveness of this treatment is small and diminishes over time [6]. Severe levels of pain intensity and disability have been identified as most prominent predictors for these disappointing results (Campbell et al., [7]; Helmhout et al., [8]). Moreover, compliance of patients with CLBP with advice and homework exercises from physiotherapists is low due to lack of patient’s motivation [9]. Also, pain related fears, including catastrophizing and fear-avoidance beliefs, might hinder patients in performance of exercises and could lead to lower adherence [8]. In addition, pain related fears are related to an increase in bodily awareness, which in turn could increase pain and pain disability [10]. By tackling these issues, physiotherapy treatment for CLBP could potentially yield better results.

To aid the physiotherapist in providing more effective treatment for patients with CLBP, virtual reality (VR) could be of use as an addition to the current primary physiotherapy care [11]. VR is a relatively new technology that has been rapidly evolving over the past years [12]. VR is defined as “the interaction between a participant and a simulated three dimensional, immersive world” [13]. Previous studies using VR as treatment modality have shown that VR could reduce pain and improve outcomes for conditions including fibromyalgia [14, 15], complex regional pain syndrome [16], chronic headache [17] and chronic neuropathic pain [18]. In regard to chronic pain, therapeutic VR has been used for several treatment goals, including distraction [19], relaxation [20], pain education [21], exposure therapy [22], activation [23] and to increase treatment motivation [24]. To sum up, VR offers the opportunity to support physiotherapists in their treatment of patients with CLPB by integrating multiple treatment goals and tailoring treatment, which coincides with the shift towards precision medicine in chronic pain [25].

Prior studies have shown that immersive VR can be successfully used in the treatment of patients with CLBP (e.g. [21, 26]). However, none of these studies specifically focused on the complex group of patients with high pain intensity and disability, even though this group seems to benefit most from treatment with VR [27]. Also, most studies do not use immersive VR as part of blended care, lack sufficiently powered samples and do not compare to a suitable control condition [28–30]. Moreover, previous studies regarding VR and chronic pain recommend long-term follow-up periods [31], an appropriate clinical setting (e.g. physiotherapy practice) [32] and possibilities to personalize the intervention [33]. These recommendations were included in the current study. Although prior studies on the effects of VR for patients with CLBP have been conducted, none of these studies deployed a pragmatic trial in which primary care physiotherapy with integrated multimodal VR was tested for patients with complex CLBP.

### Aims

The primary aim of this study is to assess the effectiveness of physiotherapy with integrated multimodal VR for patients with complex CLBP compared to usual primary physiotherapy care on physical functioning. Secondary aims include the effectiveness on pain intensity, pain related fears, pain self-efficacy, physical activity level, global perceived effect, problems with activities and the cost-effectiveness of physiotherapy with integrated multimodal VR for patients with complex CLBP, compared to usual primary physiotherapy care. This protocol describes the warranted steps to accomplish the aims of this study.

## Methods

### Design

**Fig. 1:**
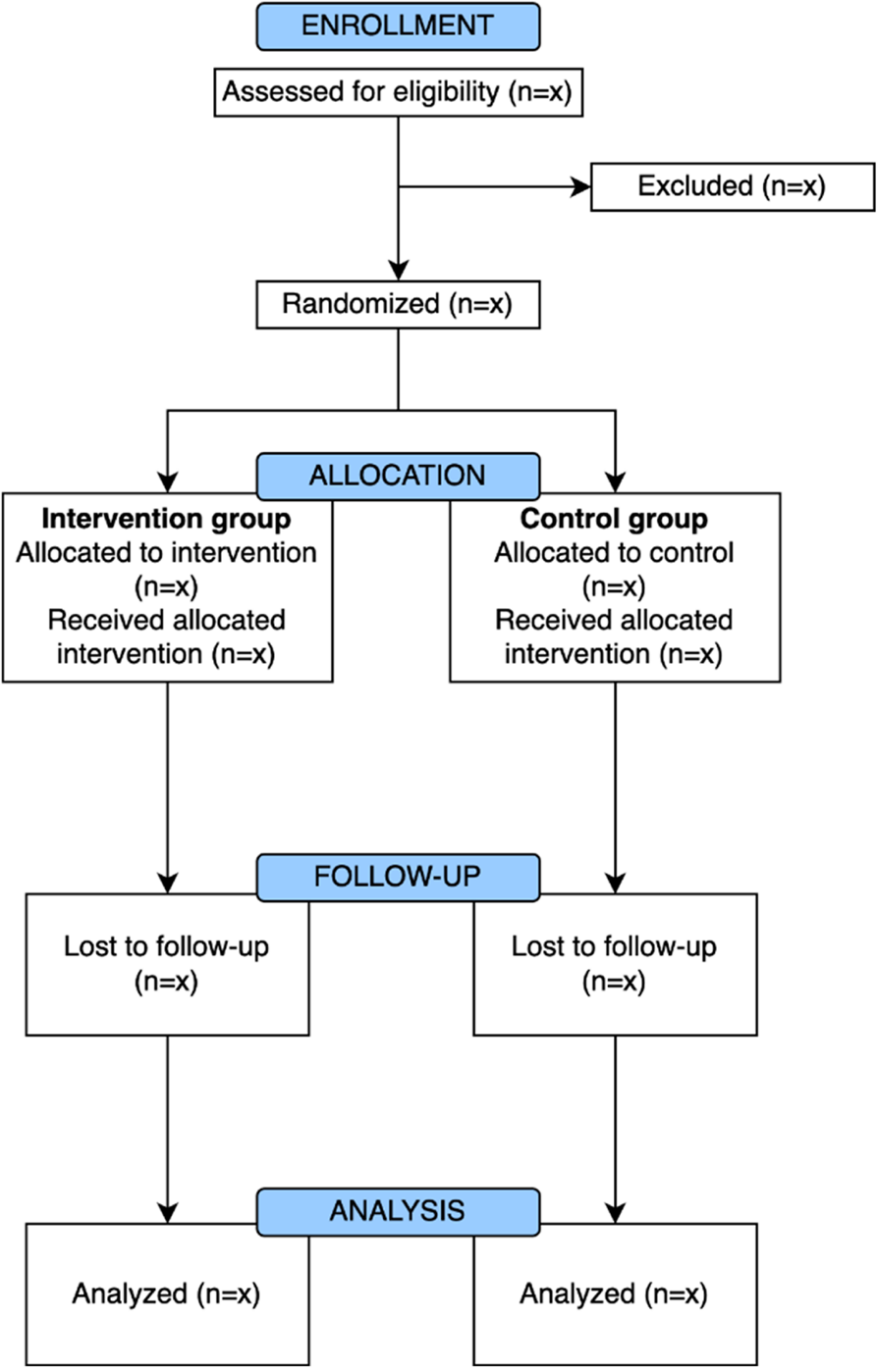
Flow of participants

This study is part of the VARIETY project and funded by ZonMw (project number: 10270032021502). A pragmatic, multicenter, two-arm, parallel, superiority, cluster randomized controlled trial (RCT) including 120 patients with CLBP from approximately 20 physiotherapists will be conducted. Physiotherapy practices (with 2–3 participating physiotherapists per practice) will be randomly allocated on a 1:1 ratio to the experimental group that will administer blended care consisting of physiotherapy treatment with integrated, immersive, multimodal, therapeutic VR, or to the control group that will administer usual primary physiotherapy care for patients with CLBP. An online software program (www.sealedenvelope.com) will be used for cluster randomization. Cluster randomization was chosen above regular randomization to prevent contamination due to physiotherapists providing both interventions. Patients will be allocated to the intervention or control group that their physiotherapy practice their physiotherapist was randomized in. The flow of participants is shown in the CONSORT flow diagram (Fig. 1) [34].

Patients and physiotherapists cannot be blinded for the treatment allocation due to the nature of this trial. On the other hand, patients will only be informed about their own intervention and will not be informed about the other arm, in line with the ethical recommendations for cluster RCTs. Physiotherapists of the control group will not be informed about the content of the experimental intervention either. An independent statistician performing the primary analyses will be blinded for treatment allocation.

In order to support best practices in the methodology of VR trials, a best practices framework consisting of three phases was developed by the VR-CORE international working group. VR1 trials focus on content development, VR2 trials conduct early pilot testing and VR3 trials use a RCT design to determine effectiveness of an intervention [32]. Current study is a VR3 trial, studying an intervention that was developed using a VR1 and VR2 trial, reported in a separate article.

This study was approved by the medical ethical review board (METC Oost-Nederland, case number: 2022–15,794) and is registered at ClinicalTrials.gov (identifier: NCT05701891).

### Participants

#### Physiotherapists

Physiotherapists (*n* = 20) will be recruited through their practices using convenience sampling through social networks (LinkedIn and Facebook) and physiotherapist networks (e.g. Dutch Society for Physical Therapy (KNGF) and local lower back pain networks). Of each responding physiotherapist practice a maximum of two physiotherapists will be included in the trial, to achieve a heterogenous sample. Physiotherapists are eligible for participation in this study if they: (1) work within 50 km from Enschede or Nijmegen in the Netherlands, (2) have no prior experience using VR as a treatment modality in physiotherapy practice, (3) treat on average at least one new patient with CLBP per month and (4) are willing to cooperate with the study protocol.

#### Patients

Participants (*n* = 120) will be newly admitted patients that visit the physiotherapist. Patients are considered to be eligible for participation if they meet the following inclusion criteria: (1) CLBP > 3 months as reason to visit the physiotherapist, (2) absence of ‘red flags’ or signs of specific LBP [5], (3) not having consulted a physiotherapist for CLBP in past 6 months, (4) combination of severe disability (Oswestry Disability Index (ODI) score ≥ 30 [35]) and severe pain (Numeric Pain Rating Score (NPRS) ≥5 [36]), (5) aged 18–80 and (6) provides informed consent. Participants will be excluded if they: (1) have severe (physical or mental) comorbidity that will substantially hinder the physiotherapy treatment, (2) have a planned diagnostic or invasive treatment procedure (e.g. injection, nerve block or operation) for their CLBP in the next 3 months, (3) lack comprehension of Dutch language, (4) are not able to use VR (e.g. epilepsy, open wounds on face or severe visual impairment) and (5) have no email address or Wi-Fi connection at home.

### Procedure

Patients visiting a participating physiotherapist practice because of CLBP are screened for study participation by the physiotherapist during the intake. If a patient is eligible, the physiotherapist provides a patient information letter and informed consent form, provides oral information about the study and asks the patient to decide within a few days to participate (and if so, provides an informed consent). In addition, the physiotherapist asks the patient’s permission to send the patient’s name and mail address to the researcher, and register this permission in their regular electronic patient file. If permitted, the physiotherapist provides the researcher the patient’s name and mail address through a fully secured app (Siilo; www.siilo.com/nl/; frequently used by general practitioners (GPs), physiotherapists and other healthcare professionals) or by calling the researcher. Subsequently, the researcher will send the first online questionnaires to the patient. If the patient decides not to participate, no questionnaires will be sent (and if applicable, the first questionnaires can be ignored), and the patient will not be refused treatment by the physiotherapist.

### Interventions

Both interventions in this trial will be 12-week physiotherapy treatments.

#### Control group

Patients in the control group will receive 8 to 24 usual physiotherapy sessions, based on the Dutch physiotherapy guidelines for CLBP [5]. The guidelines recommend pain education combined with exercise therapy, possibly complemented with behavior related treatment (e.g. graded activity, acceptance and commitment therapy (ACT) or relaxation) and non-exercise interventions (e.g. manipulations or massage).

#### Experimental group

Before the start of the trial, physiotherapists will receive a group-based training course on: (1) the developed intervention and methodology of personalizing the intervention, (2) integration of VR within physiotherapy treatment and (3) practical use of VR headsets.

The experimental intervention consists of (usual) physiotherapy with integrated VR.

#### Physiotherapy

The physiotherapy treatment will consist of 8 to 24 sessions of usual physiotherapy care as described for the control group, complemented with feedback and discussion on the patient’s VR use. Based on the patient’s experience and data on VR use, the physiotherapist can alter the treatment’s intensity, frequency and modality of (VR) treatment. Additionally, physiotherapists are encouraged to use VR for any possible homework exercises.

#### VR

In addition to the usual physiotherapy, physiotherapists in the experimental condition will integrate therapeutic VR in their treatment. Patients will be instructed to use VR on Pico Neo 3 (PICO, San Francisco, CA) head-mounted displays (HMDs), five times a week for 10 to 30 minutes at home, during the 12-week treatment period. This dosage is based on a review of CLBP trials, which found that 8–12 week interventions with a minimal duration of 20 hours were most beneficial to pain and function [37] and is congruent with the Dutch norm for physical activity. Moreover, a maximal VR duration of 30 minutes per day is advised because the risk of vertigo increases notably after this. Patients will receive instructions for operating and using the VR headset from their physiotherapist. As discussed below, patients and their physiotherapists will tailor the VR modules to the patients’ needs.

Physiotherapists and patients select which of the VR modules described below are most suited to the needs and wishes of the patient, using shared-decision making. It is advised to start the first 3 weeks of the intervention with the first two modules (Reducept and SyncVR Relax & Distract) and start with the third module (SyncVR Fit) in the fourth week. Moreover, to emphasize the importance of pain education it is advised to repeat this module in week 8 and 12, as previous research has shown that repetition is crucial in pain education treatment [38].

The following three VR modules will be used:

1. Reducept (Reducept, Leeuwarden, The Netherlands): Reducept contains five games, aiming to teach patients with CLBP that pain can be influenced and managed by changing their beliefs about chronic pain. This is done by a combination of pain education and different psychological therapies (e.g. cognitive behavioral therapy (CBT), mindfulness and visualization). Pain education has shown to be effective in the management of chronic pain, when incorporated within a multimodal therapy [39]. Psychological therapies, and mainly CBT, are also considered beneficial for patients with chronic pain [40].
2. SyncVR Relax & Distract (SyncVR, Utrecht, The Netherlands): SyncVR Relax & Distract contains 50 videos (e.g. swimming with dolphins and city tour of Amsterdam), ten games (e.g. Sudoku and Memory) and five exercises (e.g. breathing and meditation exercises). Relaxation seems to be helpful as an added intervention for patients with CLBP, especially during regular practice [41, 42]. On the other hand, distraction has great analgesic qualities, but usually works for shorter periods of time [19].
3. SyncVR Fit (SyncVR, Utrecht, The Netherlands): SyncVR Fit contains 11 movement and sports exercises, including tennis, boxing and archery. Exercise therapy is a common treatment modality in CLBP and is recommended in clinical guidelines [5, 43]. Also, patients with a higher score on pain related fears (Fear Avoidance Beliefs Questionnaire, physical activity subscale (FABQ-PA) [44] ≥16 or Pain Catastrophizing Scale (PCS) [45] ≥30) will receive additional exposure therapy by their physiotherapists, based on the following four steps: (1) mapping patient specific pain related fears, (2) education about these patients-specific pain related fears (3) facing these patients-specific fears (if possible using SyncVR Fit) and (4) facing patient-specific fears in daily life activities [46].

### Outcome measures

The time schedule of this study is shown in Table 1, following the SPIRIT recommendations [47]. The primary outcome measure is physical functioning, measured with the Oswestry disability index version 2.1a (ODI) [35]. Secondary outcome measures include pain intensity over the past week, measured using the single-item numeric rating scale (NRS) [36], pain related fears, measured using the fear avoidance beliefs questionnaire, physical activity subscale (FABQ-PA) [44] and pain catastrophizing scale (PCS) [45], general effect, measured using global perceived effect (GPE) [48], problems with activities, using the patient-specific complaints questionnaire (PSK) [49], pain self-efficacy, using the pain self-efficacy questionnaire (PSEQ) [50], intervention expectation, using the credibility and expectancy questionnaire (CEQ) [51], sense of presence, using the Igroup presence questionnaire (IPQ) [52] and physical activity, using an open-ended question. Economic measures include the quality-adjusted life years (QALYs), using the Euroqol five-dimensions (EQ-5D-5L) [53] and health-related and work-related costs, using a self-constructed questionnaire.

**Table 1:**
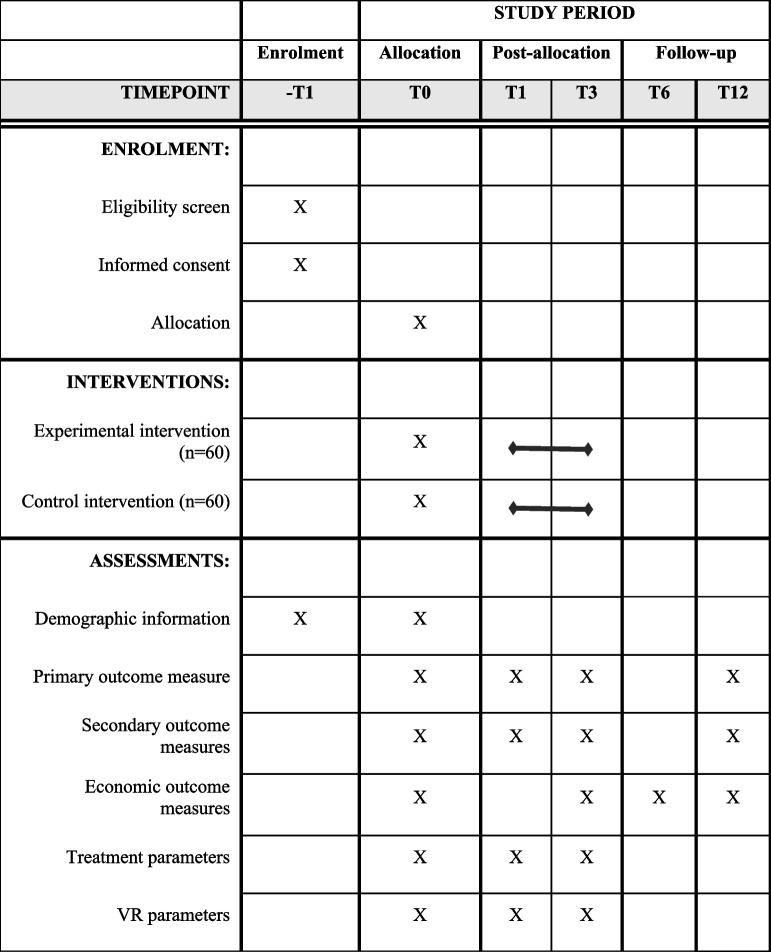
SPIRIT flowchart: time schedule of study

All questionnaires used will be in Dutch and have sufficient reliability and validity. Participants will complete questionnaires at baseline (T0), 1 month after the start of the intervention (T1), directly after finishing the intervention (T3), and follow-up after 6 months (T6) and 12 months (T12). All questionnaires will be provided to the patients using an online data management system.

Additionally, patients will be asked to report the following demographics at baseline: age, sex, BMI, tobacco use, duration of complaints, comorbidities, occupation, education level, diagnostic and therapeutic procedures in past 6 months, medication use, experience with VR for treatment and entertainment. After the intervention, patients are asked to report other treatments than physiotherapy for their CLBP in the past 12 weeks.

Physiotherapists will be asked to report their age, gender, years’ experience as a physiotherapist and specialization before the start of the trial.

The following treatment parameters will be reported by all physiotherapists after each physiotherapy treatment: modalities and duration of treatment. Additionally, physiotherapists in the experimental group will report adverse events (AEs) if necessary. These measures will give an insight in the administered physiotherapy treatment.

In the experimental group, the following VR parameters will be extracted from the online dashboard: treatment modality and duration (i.e. number of days a week and minutes per day, patients performed the modality) and VAS (before and after use of Reducept).

### Sample size

A clinically relevant between-group difference of at least 10 points on the ODI [35] at 3-months follow-up is expected, which seems plausible based on recent pilot studies with VR from our research group [33, 54] and others [27, 55–57]. For this expected and clinically relevant difference of 10 and a standard deviation of 15 (based on previous studies from our group (e.g. [58]), 60 patients are needed per group (a = 0.05; power = 90%; ICC for clusters 0.05; 15% drop-out). We expect to include 1 patient every 2 months per physiotherapist. A total of 20 participating physiotherapists will result in a total of 120 patients after 12 months.

### Statistical analysis

All statistical analyses will be done using SPSS version 27 (IBM Inc., San Diego, CA) and StataSE version 17 (Stata Corporation, College Station, TX). The statistical analysis plan (SAP) is registered at ClinicalTrials.gov (identifier: NCT05701891). The two study arms will be compared on baseline using descriptive statistics. Means and standard deviations will be presented for continuous variables (or medians and interquartile ranges in case of a skewed distribution), and frequencies and percentages for categorical variables. We will check for outliers by inspecting histograms and boxplots, the effect of any outliers will be assessed by comparing a 5% trimmed mean to the total mean. Results will be considered statistically significant if *p* < 0.05 (two-sided testing).

#### Effectiveness

Effectiveness of the experimental intervention compared to the control intervention will be analyzed on the primary outcome physical functioning. Data will be analyzed using an intention-to-treat, linear mixed-model analysis with maximum likelihood estimation (MLE), comparing both groups on clinical effectiveness at 3 months (primary end-point) and 12 months follow-up, with all follow-up time points included in the analysis. The analysis will be adjusted for major prognostic characteristics that could confound the treatment effects (e.g. pain severity), in addition to important baseline patient (e.g. age) or physiotherapist (e.g. specialization) characteristics that differ considerably between the two arms. Additionally, per protocol analyses will be carried out as described in the SAP.

Similar analyses will be performed for the secondary outcome measures pain intensity, pain related fears, pain self-efficacy, physical activity level and problems with activities. GPE will be analyzed as a dichotomous variable (i.e. completely and much recovered vs. all other responses), using logistic multilevel analysis.

#### Cost-effectiveness

Besides intervention-related costs, primary and secondary healthcare costs and medication costs will be measured. Also, costs related to presenteeism, absenteeism, unpaid productivity and informal care will be measured. Cost-effectiveness of the experimental intervention compared to the control intervention will be analyzed for the 12-months follow-up period on physical functioning and pain intensity. Cost-utility of the experimental intervention compared to the control intervention will be analyzed for the 12-months follow-up period on quality of life. All patients included in the study will be analyzed, with missing data handled by using Multivariate Imputation by Chained Equations (MICE). Costs and effect differences will be estimated using Seemingly Unrelated Regression (SUR) analyses, in which their possible correlation can be accounted for. The 95% confidence intervals surrounding the cost differences will be estimated using Bias-Corrected and accelerated (BCa) bootstrapping. Subsequently, Incremental Cost-Effectiveness Ratios (ICERs) will be estimated by dividing the differences in costs by the differences in effects. Uncertainty surrounding the incremental cost-effectiveness ratios will be graphically illustrated by plotting BCa-bootstrapped cost-effect pairs on cost-effectiveness planes. Also, cost-effectiveness acceptability curves will be constructed to provide an indication of the probability of physiotherapy with integrated multimodal VR therapy being cost-effective with usual care at different values of willingness to pay [59].

## Discussion

This protocol describes the rationale and design of a large, pragmatic, multicenter cluster randomized controlled trial, aiming to determine the clinical and cost-effectiveness of physiotherapy with integrated, personalized, multimodal, immersive VR compared to regular physiotherapy for patients with CLBP.

Opposed to most VR studies, this VR study will be a pragmatic trial in which physiotherapists will integrate VR in their treatment of patients with CLBP, instead of providing VR as standalone treatment. Another strength of this study is the thorough monitoring of patients and their treatment, including five measurement moments for patients with a maximal follow-up of 12 months. Moreover, the VR modules in this intervention are multimodal and will be personalized to the patients’ needs.

The preference for a pragmatic trial increases the external validity of the study results. However, this might to some extent compromise the internal validity [60], due to a possible range of variations in the applied interventions (e.g. duration and content of VR modules and physiotherapy sessions and expertise of physiotherapist) and a relatively small number (*n* = 10) of physiotherapists applying the experimental intervention. Moreover, due to the absence of a sham VR group, we cannot rule out a placebo effect in the experimental group.

The results of this study will contribute to the mounting literature on the effect of VR for patients with chronic musculoskeletal pain. Also, the results are interesting for practicing physiotherapists treating patients with CLBP and could aid in the discussion to add eHealth in Dutch physiotherapy care to contest the rising healthcare costs. Last and most important, the complex subgroup of CLBP patients with severe pain and severe disability might receive more effective treatment, when the VR integrated physiotherapy treatment will demonstrate superior effects.

## Data Availability

The data generated during this study will not be publicly available, but will be available upon reasonable request to the corresponding author.

## Abbreviations

ACT: Acceptance and commitment therapy
AE: Adverse event
BCA: Bias-corrected and accelerated
CBT: Cognitive behavioral therapy
CLBP: Chronic low back pain
GP: General practitioner
GPE: Global perceived effect
HMD: Head-mounted display
ICERs: Incremental cost-effectiveness ratios
LBP: Low back pain
MICE: Multivariate imputation by chained equations
MLE: Maximum likelihood estimation
SAP: Statistical analysis plan
SUR: Seemingly unrelated regression
VAS: Visual analogue scale
VR: Virtual reality
QALY: Quality-adjusted life year
